# Lentiviral vector mediated modification of mesenchymal stem cells & enhanced survival in an *in vitro *model of ischaemia

**DOI:** 10.1186/scrt53

**Published:** 2011-03-07

**Authors:** Lisa McGinley, Jill McMahon, Padraig Strappe, Frank Barry, Mary Murphy, Daniel O'Toole, Timothy O'Brien

**Affiliations:** 1 Regenerative Medicine Institute and Department of Medicine, National University of Ireland, Galway and Galway University Hospital, University Road, Galway. Ireland; 2 Multiple Sclerosis & Stroke Research Group, NCBES, National University of Ireland Galway, University Road, Galway, Ireland; 3 School of Biomedical Sciences, Charles Sturt University, Boorooma Street, PO Box 588, Wagga Wagga, NSW 2678, Australia; 4 Lung Biology Group, Department of Anaesthesia, NCBES, National University of Ireland, Galway, University Road, Galway, Ireland

## Abstract

**Introduction:**

A combination of gene and cell therapies has the potential to significantly enhance the therapeutic value of mesenchymal stem cells (MSCs). The development of efficient gene delivery methods is essential if MSCs are to be of benefit using such an approach. Achieving high levels of transgene expression for the required period of time, without adversely affecting cell viability and differentiation capacity, is crucial. In the present study, we investigate lentiviral vector-mediated genetic modification of rat bone-marrow derived MSCs and examine any functional effect of such genetic modification in an *in vitro *model of ischaemia.

**Methods:**

Transduction efficiency and transgene persistence of second and third generation rHIV-1 based lentiviral vectors were tested using reporter gene constructs. Use of the rHIV-pWPT-EF1-α-GFP-W vector was optimised in terms of dose, toxicity, cell species, and storage. The *in vivo *condition of ischaemia was modelled *in vitro *by separation into its associated constituent parts i.e. hypoxia, serum and glucose deprivation, in which the effect of therapeutic gene over-expression on MSC survival was investigated.

**Results:**

The second generation lentiviral vector rHIV-pWPT-EF1-α-GFP-W, was the most efficient and provided the most durable transgene expression of the vectors tested. Transduction with this vector did not adversely affect MSC morphology, viability or differentiation potential, and transgene expression levels were unaffected by cryopreservation of transduced cells. Over-expression of HSP70 resulted in enhanced MSC survival and increased resistance to apoptosis in conditions of hypoxia and ischaemia. MSC differentiation capacity was significantly reduced after oxygen deprivation, but was preserved with HSP70 over-expression.

**Conclusions:**

Collectively, these data validate the use of lentiviral vectors for efficient *in vitro *gene delivery to MSCs and suggest that lentiviral vector transduction can facilitate sustained therapeutic gene expression, providing an efficient tool for *ex vivo *MSC modification. Furthermore, lentiviral mediated over-expression of therapeutic genes in MSCs may provide protection in an ischaemic environment and enable MSCs to function in a regenerative manner, in part through maintaining the ability to differentiate. This finding may have considerable significance in improving the efficacy of MSC-based therapies.

## Introduction

Mesenchymal stem cells (MSCs) represent a significant area of interest in the field of cell therapy. They are a multipotent, self-renewing cell population isolated from bone marrow, capable of differentiation into cells of different lineages including chondrocytes, osteoblasts and adipocytes [1]. The involvement of MSCs in tissue repair, their ability to home to sites of injury after systemic delivery [2,3] and their evasion of normal host immune responses [4,5] make them an attractive cell type for transplantation into various disease models. MSCs have also generated considerable attention in recent years in the gene therapy area, as possible vehicles for the delivery of therapeutic genes to sites of injury. Genetic modification of MSCs may allow further improvement of their therapeutic potential through promotion or suppression of gene expression. An additional benefit of MSCs as a gene delivery system is their lack of immunogenicity, which would confer significant advantage to gene therapy techniques, as the MSC itself may prevent a vector-induced host immune response [5-7].

Current cell-based therapeutic approaches for cardiovascular injury and disease are promising, although not without their shortcomings. MSC transplantation to the infarcted heart aims to promote repair and regeneration of the damaged cardiac tissue. Although several studies have proved somewhat successful [8], it may be possible to equip the MSCs for the *in vivo *environment into which they will be transplanted. Conditions of hypoxia, ischaemia and oxidative stress are associated with the infarct and its surrounding environment. *In vitro *studies have shown that, in the absence of oxygen, MSCs are capable of survival for a limited time [9]. A more prolonged lifespan may be necessary for MSCs to exert a desired therapeutic effect *in vivo*. In this respect, we hypothesize that increasing MSC survival capacity by genetic modification could afford the MSC a longer time in which to mediate repair and recovery both by direct and paracrine means, thereby enhancing their *in vivo *therapeutic value.

MSCs have been modified by various methods including lipid transfection and viral transduction. It is necessary to tailor the vector type chosen for MSC modification to the specific applications for which the cells are to be used. Lipid transfection of MSCs results in relatively low numbers of transgene expressing cells, approximately 30-40% maximum [10]. Modification with viral vectors generally results in higher efficiency that may express transgene long or short term, depending on choice of vector. Our group has previously published a study comparing various *in vitro *modification methods for rat MSCs. It was reported that lentiviral vectors demonstrated excellent transduction of rat MSCs in contrast to adenovirus, AAV, lipid transfection and electroporation [11]. Accurate knowledge of gene transfer performances, in particular vector and promoter efficiency, is central to paving the way to preclinical and eventually human trials.

Lentiviral vectors have long since been established as having the ability to transduce a broad range of cell types efficiently, including non-dividing, senescent and terminally differentiated cells [12,13]. Lentiviral vectors integrate into the host cell genome resulting in stable long-term transgene expression. Elements to be considered upon choosing a vector type include vector generation, vector envelope and promoter type. An additional important consideration with regard to viral vector gene delivery is the possibility of epigenetic transgene suppression or inactivation by target host cells after transduction has occurred. This is of particular concern, as its occurrence may adversely affect the use of viral vectors for *ex vivo *stem cell modification.

The present study aims to assess lentivirus vector-mediated genetic modification of MSCs, specifically transduction efficiency, durability of transgene expression and transgene or promoter "silencing" of three different lentiviral vector constructs. In addition, the effect of lentiviral vector transduction on MSC viability and differentiation capacity was examined. The effects of cell cryopreservation, cell passage number and species on transgene expression were also assessed. Further to establishing an optimal vector system for the modification of MSCs, a second objective of this study was to gain an understanding of how MSCs react to and influence post-ischaemic recovery, and to ascertain if this can be enhanced by induced therapeutic antioxidant or anti-apoptotic gene over-expression. This series of experiments included an evaluation of the survival and differentiation potential of MSCs in an *in vitro *model of ischaemia and its components, the main goal being to determine whether lentivirus vector-mediated over-expression of the pro-survival proteins catalase, SOD1, SOD3 and HSP70 can facilitate increased MSC survival.

## Materials and methods

All reagents utilised were from Sigma-Aldrich (St. Louis, MO, USA) unless otherwise stated.

### Lentivirus production & titration

The present study compared second and third generation self-inactivating (SIN) human immunodeficiency virus-1-based (HIV-1), VSV-G pseudotyped lentiviral vectors. Tat-dependent second generation lentiviral vectors consisted of rHIV-pWPT-EF1-α-GFP-W and rHIV-cPPT-CMV-GFP-W. The associated second generation packaging system production plasmids were provided by Didier Trono (Lausanne, Switzerland). Vectors were produced by standard transient transfection of a three-plasmid system into producer cells. Briefly, packaging plasmid ps-PAX2.2 (Addgene plasmid 12260), envelope plasmid pMD2.G (Addgene plasmid 12259) and either pWPT-GFP (Addgene plasmid 12255) or cPPT-CMV-GFP expression plasmids were transfected into HEK293 cells using Jet PEI transfection reagent (Polyplus Transfection Inc, NY, USA) according to manufacturer's instructions. Culture medium was replaced 16 h post-transfection. Vector-containing supernatants were collected 48 h and 72 h post-transfection, filtered, pooled and concentrated by ultracentrifugation at 27,000 × g for 3 h. Lentivirus vector titre was determined by a quantitative real-time PCR based method, which detected stably integrated virus sequences in target HeLa cells and was expressed as transducing units per ml (TU/ml). Tat-independent third generation lentivirus vector, PPT-PGK-GFP-WPRE, containing the WPRE element and polypurine tract with the GFP transgene under the control of the PGK promoter was produced by Genethon (Genethon, Évry Cédex, France, ref. pG3.22116) using four-plasmid transfection of 293T cells. Human Catalase, HSP70, SOD1 and SOD3 genes were cloned into the lentiviral expression plasmid pWPT using *MluI *and *SalI *restriction sites and lentiviral vectors expressing these transgenes were produced and vector titre calculated as described above.

### Human mesenchymal stem cell isolation and expansion

Marrow was obtained from donors after informed consent and all procedures were approved by the Clinical Research Ethics Committee at University College Hospital (Galway, Ireland). Briefly, 30 ml heparin-treated bone marrow aspirates were acquired in separate 6 ml aliquots from the iliac crest of healthy donors. Aliquots were pooled, washed in PBS and cell pellets were resuspended in 10 ml of complete human MSC medium, which consisted of low glucose DMEM, 10% (v/v) FBS and 1% penicillin/streptomycin mix. Cells were seeded into 175 cm^2 ^flasks at a density of 5.7 × 10^5 ^cells per cm^2^. After 5 days, non-adherent red blood cells were removed by a PBS wash and cells were re-fed with 30 ml of complete medium per flask. When MSC colonies had reached 80-90% confluence, cells were detached with 0.25% trypsin(w/v)/1 mM EDTA solution, re-plated at 5.7 × 10^3 ^cells per cm^2 ^with subsequent subculture, usually at 4-6 day intervals.

### Rat mesenchymal stem cell isolation and expansion

All procedures involving animals were performed in accordance with the ethical regulations of the National University of Ireland (Galway, Ireland). MSCs were isolated from the bone marrow of 8-12 weeks old male Sprague Dawley rats (Harlan Laboratories, Oxfordshire, UK) as previously described [14-16]. Briefly, after euthanasia, marrow was flushed from femoral and tibial compartments with complete rMSC growth medium, which consisted of 44.5% α-MEM (Gibco, Invitrogen, Carlsbad, CA, USA) and 44.5% F12-Ham (Gibco), supplemented with 10% FBS (Gibco) and 100 U/ml penicillin-G and 100 μg/ml streptomycin sulphate (Gibco). Recovered suspensions were pooled, counted and plated at a density of 1.2 × 10^6 ^cells per cm^2^. Non-adherent cells were removed after 3 days and cells were re-fed with complete rMSC medium, with additional media changes every 3-4 days. After approximately 8 days or when cell cultures reached confluence, cells were detached with 0.25% trypsin(w/v)/1 mM EDTA solution and re-plated at 5.7 × 10^3 ^cells per cm^2^, with subsequent passage when they again reached confluence.

### MSC characterisation

The ability to differentiate down the adipogenic, osteogenic and chondrogenic lineages, following isolation procedures described, was routinely tested in-house. According to criteria recommended by the International Society for Cellular Therapy (ISCT), an MSC deemed acceptable for laboratory based investigations and pre-clinical studies is defined by its adherence to plastic, differentiation capacity and cell surface markers (CD105, CD73, CD90 positive and CD34, CD45, CD14 or CD11b, CD79a or CD19 and HLA-DR negative). Isolated MSCs were routinely characterised in-house by flow cytometry for their cell surface markers, as previously described [10,11].

### MSC transduction and gfp reporter gene detection

MSCs were seeded in 6-well plates at 1 × 10^5 ^cells per well, grown overnight and transduced with lentiviral vectors in a minimal volume of medium. MSCs were harvested for analysis, at the earliest, 96 h post-transduction. For the 28-day transgene analysis experiments, cells were harvested at 7 d intervals, at which point each cell population per well was split, using one half to maintain the cells in culture and the other for GFP expression analysis (see below). For assessment of transgene silencing, transduced cell populations were cultured in medium containing the histone deacetylase (HDAC) inhibitor, trichostatin A (TSA; 100 nM) and/or the DNA methyltransferase inhibitor 5-azacytidine (5-aza-c; 5 μM) for the 28-day period, harvesting at 7 d intervals. MSCs were harvested by trypsinisation, fixed in 4% paraformaldehyde and resuspended in PBS. Percentage GFP-positive cells and their mean fluorescence intensity (MFI) was determined using Guava Express Plus analysis on a GuavaEasyCyte™ flow cytometer (Guava Technologies, Millipore, Billerica, MA, USA). Total GFP was calculated by multiplication of the percentage GFP positive cells by the MFI.

### Therapeutic transgene expression analysis

Rat MSCs were transduced by incubation with lentiviral vectors at a multiplicity of infection (MOI) of 100 for 18 hours. Transgene expression was measured 72 h post-transduction by immunofluorescent staining and Western blot analysis (Figure S1 in Additional file 1).

### *In vitro *ischaemia treatments

To mimic the *in vivo *scenario of ischaemia, MSC were exposed to conditions of hypoxia (O_2 _at 0.5% plus complete DMEM), ischaemia (O_2 _at 0.5% in serum and glucose free DMEM) and complete O_2 _and glucose deprivation by inhibition of glycolysis (ischaemia+2-deoxyglucose) in an *in vivo 400 *hypoxia chamber (Ruskinn Technologies, UK). 2-deoxyglucose (2DG) is a glycolytic inhibitor, which prevents hexokinase phosphorylation of glucose. Addition of this inhibitor provided an *in vitro *model of complete glucose deprivation. For these experiments, MSCs were seeded and transduced under normal culture conditions until 72 h post transduction when cells were optimally expressing transgene. The appropriate media for each condition (hypoxia, ischaemia, ischaemia + 2DG) was placed in the hypoxia chamber for a minimum of 3 hours to deplete the oxygen levels to the required 0.5%. Depleted media was added to MSCs, which were treated with the appropriate conditions for the required period of time.

### Assessment of viability and apoptosis

MSC viability was determined by standard MTT assay as described previously [17]. Apoptosis levels were assessed by identification of apoptotic nuclei by DAPI staining of MSCs. Percentage apoptotic cells was determined in a semi-quantitative manner by counting numbers of apoptotic nuclei per field of ten random images. Caspase-3 activation was also examined using Ac-DEVD-AFC substrate (Biomol, Enzo Life Sciences, PA, USA). Staurosporine (STS - at 500 nM for 12 h) was used as a positive control for caspase activation.

### Adipogenesis differentiation assay

MSCs (both transduced and non-transduced) were seeded at 2 × 10^5 ^cell per well in 6-well plates. Once cells had reached confluence, adipogenic differentiation was induced by three 72 h cycles of adipogenic induction media (1 μM dexamethasone, 10 μg/ml insulin, 200 μM indomethacin, 500 μM 3-isobutyl-1-methyl-Xanthine, 100 U/ml penicillin, 100 μg/ml streptomycin, 10% FBS and 5% rabbit serum in high-glucose DMEM). Following each round of induction, cells were maintained in maintenance medium for 24 h (10 μg/ml insulin in complete high glucose medium), and for 5-7 days after final induction. Cells were fixed in 10% formalin and differentiated cells were identified by Oil Red O stain for lipid vacuoles. Images of 10 random fields for each well were acquired and differentiated cells containing stained lipid vacuoles per microscope field were counted. Oil Red O stain was also quantified by extraction of the stain into 99% isopropanol and absorbance measurement on a Wallac plate reader at 490 nm. Non-induced MSCs, maintained in normal growth medium, were included as negative controls for the adipogenesis assay.

### Statistical analysis

Data are presented as mean ± standard deviation (*n *= 3) of three independent experiments. Statistical analyses were carried out on data sets using SigmaStat for Windows, version 3.5. One-way analysis of variance (ANOVA) was used to determine statistical significance between groups, followed by multiple paired comparisons for normally distributed data (Tukey Test). Non-normally distributed data were analysed by ANOVA on ranks using either Dunn's Test or Kruskal-Wallis. Paired t-tests were used for pairwise comparisons. Values of p < 0.05 were considered statistically significant (*p < 0.05, **p < 0.001).

## Results

### Lentiviral vector efficiency in rat MSCs

**Figure 1:**
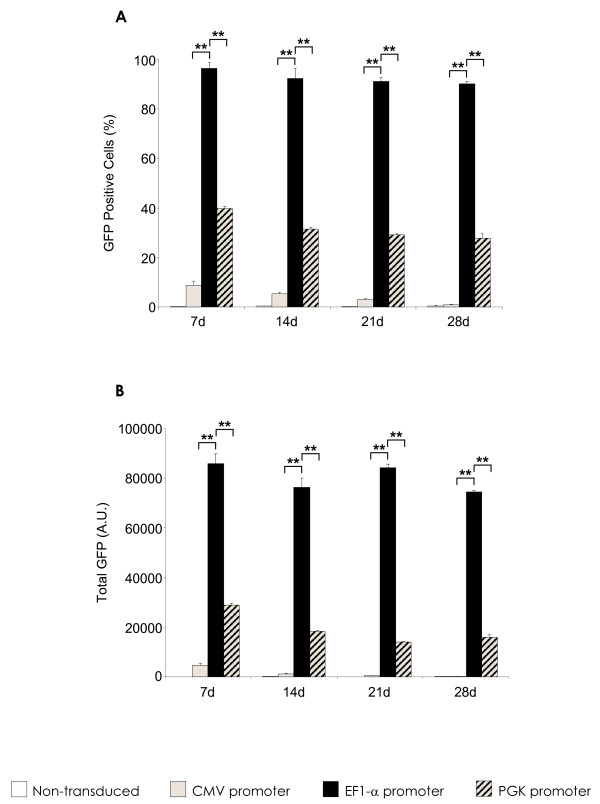
**Lentivirus vector efficiency in rat MSC**. **(A) **Percentage GFP positive cells and **(B) **total GFP (mean fluorescent intensity X % GFP positive cells) in MSCs after transduction with the described lentivirus vectors over a 28 day period with no selection methods at MOI 100. Data are shown as mean ±SD (*n *= 3) of three independent experiments. *p < 0.05, **p < 0.001 rHIV-pWPT-Ef1-a-GFP-W vector vs. PGK and CMV promoter-containing vectors.

Of the vectors evaluated, it was determined that the second generation VSV-G pWPT-EF1-α-GFP-W was the most efficient at rat MSC transduction. At MOI 100, this vector gave transduction efficiencies of 90-98% at 7, 14, 21 and 28 days respectively (Figure 1A), and total GFP values of over 75,000 at all time points (Figure 1B). This vector consistently mediated high level, sustained GFP transgene expression levels with no significant reduction throughout the time frame analysed. This was in contrast to the rHIV-cPPT-CMV-GFP-W and rHIV-PPT-PGK-GFP-W lentiviral vectors, which were tested in parallel, and consistently gave percentage GFP positives of 1-5% and 35-40% (Figure 1A), and total GFP values of 4,552-186 and 28,866-15,806 (Figure 1B) respectively. These significantly lower values were observed at all MOIs tested (data not shown).

### Lentiviral vector transgene silencing in rat MSCs

**Figure 2:**
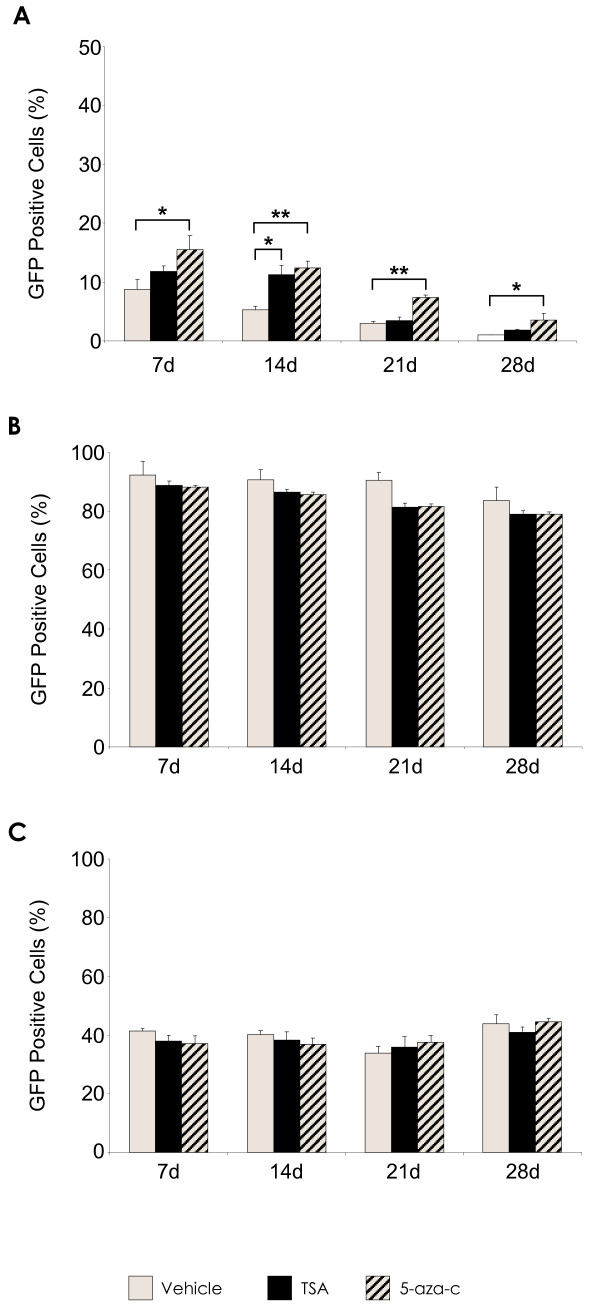
**TSA/5-AZA-C treatment on GFP transgene expression in lentiviral vector modified rat MSCs over 28d**. **(A) **rHIV-cPPT-CMV-GFP-W transduced rMSC at MOI 100. **(B) **rHIV-pWPT-EF1-a-GFP-W transduced rMSC at MOI 100. **(C) **rHIV-PPT-PGK-GFP-W transduced rMSC at MOI 100. Data are shown as mean ±SD (*n *= 3) of three independent experiments. *p < 0.05, **p < 0.001 vs. no drug.

Silencing of vector transgene is an important factor to consider where long term stable expression of transgene is required in target cells. Here, the reactivation potential of reporter transgene was assessed over a 28-day time period, by treatment of transduced cells with the HDAC inhibitor TSA, and the DNA methyltransferase inhibitor 5-aza-c. The existence of vector silencing was indicated by an increase in reporter gene expression detectable by FACS, following release of promoter function after drug treatment (Figure 2). TSA and 5-aza-c treatment of rHIV-cPPT-CMV-GFP-W transduced rMSCs resulted in significant increases of GFP positive cells at an MOI of 100, indicating that reporter transgene may have been reactivated upon exposure to the methylation and deacetylation inhibitors (Figure 2A). TSA exposure increased the percentage of GFP positive cells at all time points, these increases were significant at day 14 and day 28 (p < 0.05). At all measured time points, treatment with 5-aza-c significantly increased GFP transgene expressing cell numbers compared to non-treated control cells, with the largest observed increases at day 14 and day 21 (p < 0.001). Although there were increases in rHIV-PPT-PGK-GFP-W vector transduction levels (Figure 2B), these were not significant compared to controls. Likewise, there were no apparent changes in transgene expression levels of rHIV-pWPT-EF1-α-GFP-W transduced MSCs (Figure 2C), which suggests that transgene silencing is not a significant issue with respect to these particular constructs. Similar results were observed at other MOIs tested (data not shown).

### Transduction with second generation pWPT-EF1-α-GFP-W lentiviral vector does not affect viability or differentiation potential

**Figure 3:**
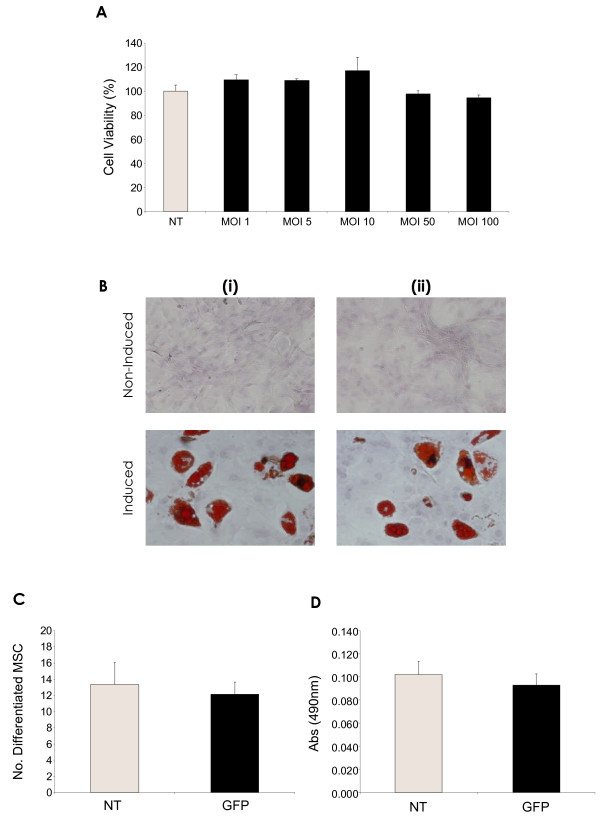
**rHIV-pWPT-EF1-a-GFP-W lentiviral vector modification does not affect msc viability or differentiation potential**. **(A) **Evaluation of rHIV-pWPT-EF1-a-GFP-W vector toxicity on rMSC viability by MTT assay. **(B) **Representative images (magnification 20X) of non-induced control MSCs and MSCs induced towards the adipogenic pathway (i) non-transduced, (ii) rHIV-pWPT-EF1-a-GFP-W transduced (MOI 100) MSCs. **(C) **Oil red O positive cells per microscope field (number of differentiated cells). **(D) **Oil red O quantitation. Data are presented as mean ±SD (*n *= 5) or as representative images of three independent experiments.

Toxicity of the vectors, following transduction was analysed by measurement of cell viability, using MTT assay (Figure 3A). No significant adverse effect on cell viability was apparent when compared to non-transduced control cells. Increase of viral dose resulted in an expected increase in cell death, but these numbers always remained low, with a percentage cell death of only ~5% being seen at the maximal MOI of 100.

MSC differentiation capacity was determined by lipid vacuole formation after a 21-day adipogenesis assay (Figure 3B,C,D). Under normal culture conditions, MSCs transduced with the rHIV-pWPT vector underwent adipogenesis at the same efficiency as control non-transduced MSCs (Figure 3B,C,D). Inhibitory effects on rat MSC adipogenic differentiation ability were not observed, either in terms of numbers of differentiated cells (Figure 3C) or amount of lipid vacuoles measured spectrophotometrically (Figure 3D).

### Factors affecting transgene expression in pWPT-EF1-α-GFP-W modified MSCs

**Figure 4:**
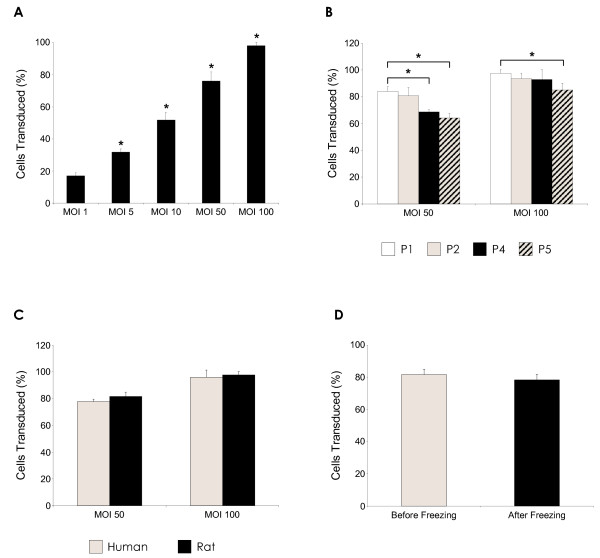
**Factors affecting transgene expression in rHIV-pWPT-EF1-a-GFP-W vector modified MSCs**. **(A) **Significant dose response (*p < 0.05 vs. MOI0) with increasing MOI (1-100) following rHIV-pWPT-EF1-a-GFP-W transduction of rMSC. **(B) **Effect of rMSC passage number on lentiviral vector transduction efficiency (*p < 0.05 vs. P1 cells). **(C) **Comparison of lentiviral vector transduction of human and rat MSCs. **(D) **Effect of cryopreservation on GFP transgene expression levels. Data are shown as mean ±SD (*n *= 3) of three independent experiments.

A significant dose response, with increasing virus particle concentration from MOI 1 to MOI 100 (p < 0.05), was achieved in rat MSCs transduced with rHIV-pWPT-EF1-α-GFP-W vector, as expected. The percentage of GFP-positive MSCs increased from ~17% at MOI 1 to ~98% at MOI 100 (Figure 4A). The effect of MSC passage number on pWPT-Ef1-α-GFP-W transduction efficiency and transgene expression was analysed by transduction of rat MSCs of early and late passage numbers and determination of numbers of transduced cells by FACS analysis. Here, a difference in transgene expression levels between the passage numbers was apparent (Figure 4B). At both MOIs measured, there were significant differences between early and late MSC passage numbers. At MOI 50, there was a decrease of approximately 15% in P5 and 13% in P4 MSCs compared to P1 MSC populations (p < 0.05). At the higher MOI of 100, a similar decrease in percentage transduced cells with increasing passage number was observed, however this was only significant between P1 and P5 MSC populations, with a reduction of approximately 8% (p < 0.05). A direct comparison was made between rat and human MSCs, which had been transduced at MOI 50 and MOI 100 by pWPT-EF1-α-GFP-W lentiviral vector (Figure 4C). There was no significant difference found between transduction levels in human and rat MSCs, at either MOI tested. The effect of cryopreservation on transduced rMSC populations was also examined (Figure 4D), where no negative impact on transgene GFP expression was detected.

### Hypoxia

**Figure 5:**
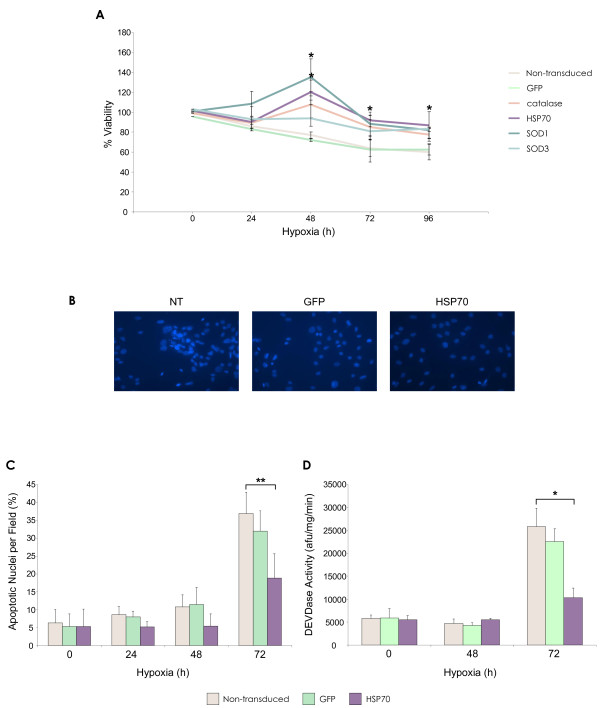
**Enhanced MSC survival by lentiviral vector modification in hypoxia**. **(A) **Percentage rMSC viability as determined by MTT assay after hypoxia exposure (*p < 0.05 HSP70-MSCs and SOD1-MSCs vs. non-transduced MSCs). **(B) **DAPI staining of MSCs after 72 h hypoxia. **(C) **Percentage apoptotic nuclei per microscope field (*n *= 10) at 72 h hypoxia (**p < 0.001 HSP70 MSCs vs. non-transduced MSCs). **(D) **Caspase-3 activity in MSC after 48 h and 72 h hypoxia (*p < 0.05 HSP70-MSCs vs. non-transduced MSC). Data are shown as mean ±SD (*n *= 3) or as representative images of three independent experiments.

MSC viability was detected by MTT assay after exposure to hypoxic conditions (Figure 5A). MSC viability decreased with increasing hypoxia exposure time, from approximately 97% at 12 hours of hypoxia to ~60% at 96 h. Transduction with the HSP70 lentiviral vector, and consequent HSP70 over-expression, significantly increased percentage viability at both 72 h and 96 h of hypoxia compared to non-transduced and GFP control MSCs (p < 0.05). Catalase and SOD over-expression did not increase viability to the same extent as HSP70, although at 48 h SOD1-MSCs were more viable than controls (p < 0.05). Control GFP MSC percentage viability remained similar to that of non-transduced MSCs, proving that any observed effects were as a result of transgene over-expression and not due to the lentiviral vector itself.

The effect of HSP70 over-expression on hypoxia-induced apoptosis was also assessed. Classical signs of apoptotic cell death were visible with respect to nuclear morphology, which included misshapen or "bean-shaped" nuclear membranes, intense fluorescence indicative of nuclear condensation, nuclear fragmentation and disassembly into apoptotic bodies (Figure 5B). The percentage of apoptotic cells was also calculated from these images (Figure 5C). A significant increase in apoptotic nuclei was evident after 72 h of hypoxia, at which point numbers were significantly reduced in the HSP70 group compared to controls (p < 0.001). The morphological changes indicative of apoptosis were confirmed by caspase activity measurement at 48 and 72 hours of hypoxia (Figure 5D). At 48 h there was no apparent increase in caspase activity in either the non-transduced or transduced MSC groups. At 72 h, there was a significant increase in caspase activity across the groups, which was significantly reduced in transduced MSCs that were over-expressing HSP70, compared to control MSCs (p < 0.05).

### Ischaemia

**Figure 6:**
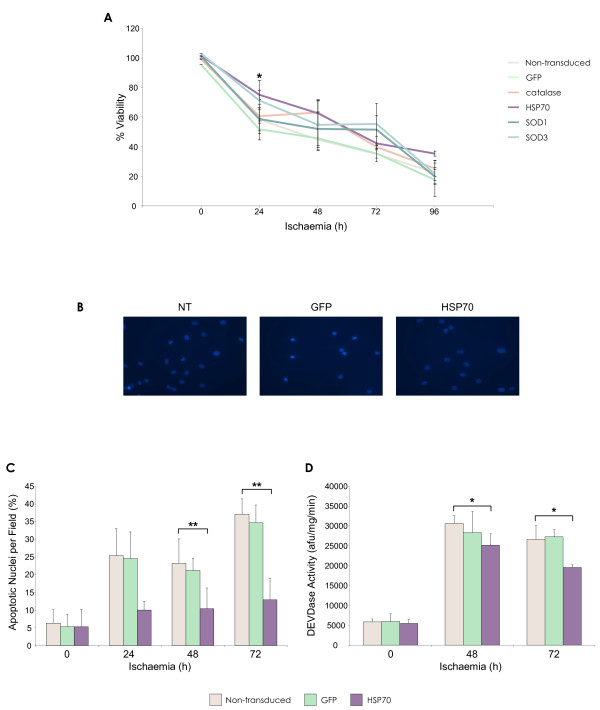
**Enhanced MSC survival by lentiviral vector modification in ischaemia**. **(A) **Percentage rMSC viability as determined by MTT assay after ischaemia exposure (*p < 0.05 HSP70-MSCs vs. non-transduced MSC). **(B) **DAPI staining of MSCs after 72 h ischaemia. **(C) **Percentage apoptotic nuclei per microscope field (*n *= 10) (**p < 0.001 HSP70-MSCs vs. non-transduced MSCs at 48 h and 72 h ischaemia). **(D) **Caspase-3 activity in rMSC after 48 h and 72 h ischaemia (*p < 0.05 HSP70-MSCs vs. non-transduced MSCs). Data are shown as mean ±SD (*n *= 3) or as representative images of three independent experiments.

Not unexpectedly, MSCs were more sensitive to conditions of ischaemia than hypoxia (Figure 6A). After 12 hours of ischaemia, cell viability had decreased to approximately 70%, falling to 20% by 96 h. Exogenous expression of HSP70 produced a significantly higher percentage of viable MSCs at several time points compared to control groups at 96 h (p < 0.05). No significant increases in viability were measured for catalase or SOD modified MSCs compared to controls at any time point. An increase in apoptotic nuclei number was apparent as ischaemia exposure time increased (Figure 6B). Fewer apoptotic nuclei were present in the HSP70 group at 48 h and 72 h (Figure 6C), which was significant compared with controls (p < 0.001). Increased caspase-3 activity was observed across all MSC groups exposed to ischaemia at the 48 h and 72 h time points examined (Figure 6D). Caspase activity was significantly decreased in HSP70-MSCs compared to non-transduced and GFP-MSC controls (p < 0.05).

### Ischaemia and 2-deoxyglucose (2DG)

**Figure 7:**
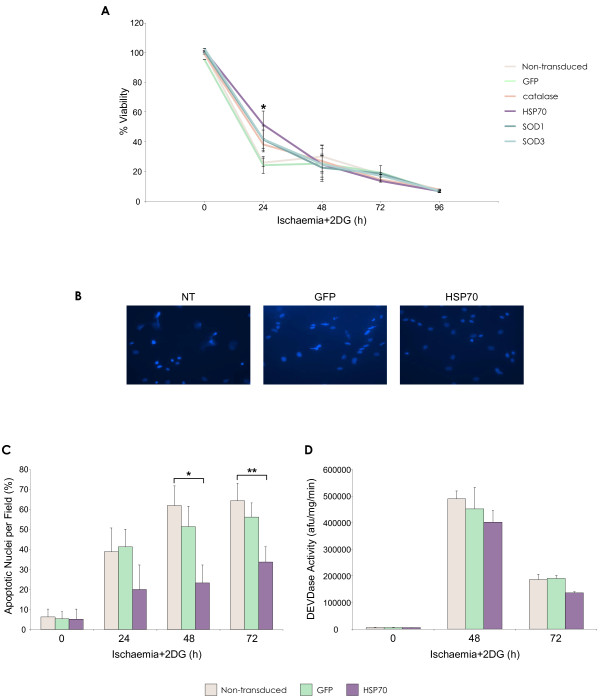
**Enhanced MSC survival by lentiviral vector modification in ischaemia with glycolysis inhibition - complete glucose deprivation**. **(A) **Percentage rMSC viability as determined by MTT assay after ischaemia with 2DG exposure (*p < 0.05 HSP70-MSC vs. non-transduced MSC). **(B) **DAPI staining of MSCs after 72 h ischaemia + 2DG. **(C) **Percentage apoptotic nuclei per microscope field (*n *= 10) (*p < 0.05, **p < 0.001 HSP70-MSCs vs. non-transduced MSC). **(D) **Caspase-3 activity in MSC after 48 h and 72 h ischaemia + 2DG (*p < 0.05 HSP70-MSCs vs. non-transduced MSC). Data are shown as mean ±SD (*n *= 3) or as representative images of three independent experiments.

To mimic the *in vivo *scenario of ischaemia where cells may not be able to use glycolysis for ATP level maintenance, MSCs were treated with 1 mM 2DG in addition to the conditions of ischaemia previously described. Addition of 2DG exacerbated the observed effects on MSC survival in terms of viability and apoptosis. After 12 hours of treatment, viability of unmodified MSCs had decreased below 40%, falling to less than 10% at the latest time point examined (Figure 7A).

At the early time points of 12 and 24 hours, the HSP70-MSC group displayed significantly increased viability, compared to controls at 24 h (p < 0.001). There was no apparent effect of exogenous HSP70 expression at any later time points. Analysis of nuclear morphology showed that increased numbers of apoptotic cells were present with ischaemia + 2DG exposure compared to the previous treatments (Figure 7B). Numbers of apoptotic nuclei were significantly reduced in the HSP70 group compared to controls at 48 h (p < 0.05) and 72 h (p < 0.001) (Figure 7C). Caspase 3 activity levels were stimulated by ischaemia 2DG treatment and peaked at 48 h, after which activity levels dropped by almost half (Figure 7D). At both time points examined, induced HSP70 over-expression did not offer significant protection against caspase-3-dependent apoptosis.

### Hypoxia, ischaemia and MSC differentiation

**Figure 8:**
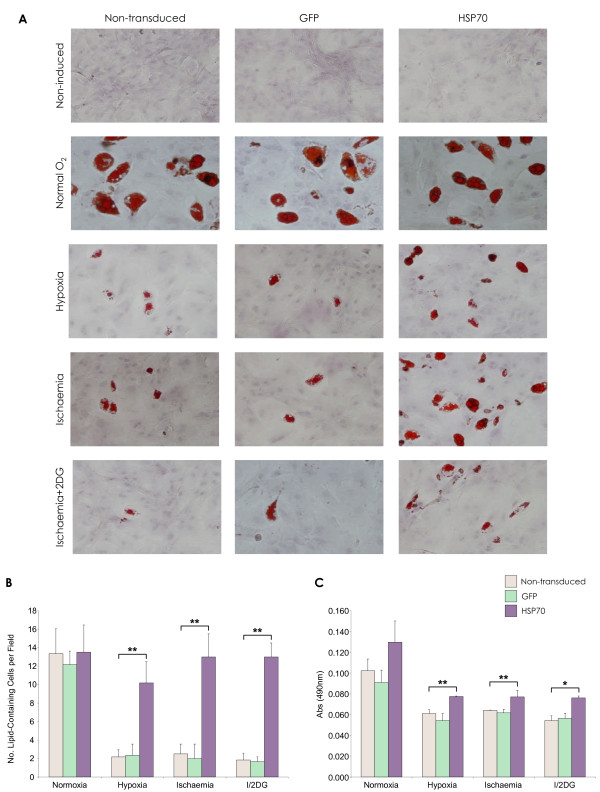
**Enhanced MSC differentiation by lentiviral vector modification in hypoxia and ischaemia**. **(A) **Oil Red O staining for lipid vacuoles post rMSC adipogenic differentiation. There was no negative effect of lentivirus vector transduction and over-expression of GFP or HSP70 on MSC adipogenic differentiation capacity. **(B) **Numbers of lipid-containing cells per microscope field (*n *= 10) (**p < 0.001 HSP70-MSCs vs. non-transduced MSC). **(C) **Oil Red O quantity (*p < 0.05, **p < 0.001 HSP70-MSCs vs. non-transduced MSC). Data are shown as mean ±SD (*n *= 3) or as representative images of three independent experiments.

The effect of ischaemic stress on MSC differentiation capacity was determined by lipid vacuole formation after a 21-day adipogenesis assay. Differentiated cells were identified by Oil Red O staining for lipid vacuoles (Figure 8A). MSCs that were exposed to *in vitro *models of ischaemia produced fewer lipid vesicles compared to normoxic MSCs. However, HSP70 over-expression reduced this inhibitory effect of oxygen, serum and glucose deprivation on MSC adipogenesis. This was in terms of numbers of differentiated cells (Figure 8B) and the amount of lipid vacuoles measured (Figure 8C), compared with controls (p < 0.05, p < 0.001).

## Discussion

### Genetic modification of MSCs

Numerous studies have utilised genetically engineered MSCs, which have been modified by various gene therapy vectors including adenovirus [18,19], AAV [20], non-viral vectors [21,22], and lentivirus [23,24]. Initial vector transduction efficiency and optimisation studies are the foundation of future *in vivo *experiments and therefore the efficiency of vector transduction of MSCs determines whether a particular preclinical model is worth pursuing. In the present study, we compare three different lentiviral vector types; two second generation vectors containing either the EF1-α or CMV promoter and a third generation vector with GFP transgene expression under control of the PGK promoter. In the second generation lentiviral vector system, five of the nine HIV-1 genes are eliminated, retaining genes for virion structural proteins and enzymes (gag/pol) and genes relating to transcriptional and post-transcriptional functions (tat and rev). These vectors are produced with a 3 plasmid packaging system. The latest third generation vectors have further HIV-1 genes deleted leaving only gag, pol and rev and are produced using a four-plasmid set. This vector generation has been designed using a chimeric LTR (long terminal repeat) ensuring transcription in the absence of tat. As a result, this vector system possesses a higher safety profile and is more suitable for clinical applications. Vector generation and promoter type will undoubtedly affect transduction levels - low or short-lived transgene expression levels, cytotoxicity or transgene silencing may prompt the choice of an alternative vector type or model. Consequently, a full investigation into the *ex vivo *transduction potential of MSCs derived from the same species is essential. This series of experiments set out to assess and optimise lentiviral vector transduction efficiency in rat MSCs, and also to examine any functional effect of genetic modification in an *in vitro *model of ischaemia.

Gene silencing can present problems when using gene delivery as certain vectors and promoters have been shown to be susceptible to transcriptional silencing in particular host cell types. It is widely accepted that retroviruses are silenced in stem cells [25-28], however, it remains controversial as to whether HIV-1-based SIN lentiviral vectors are also subjected to the same effects [29-32]. There have been several reports that SIN lentiviral vectors can be silenced to some degree in embryonic stem (ES) cells [29] and primary murine hematopoietic stem cells (HSCs) [32], with varying silencing rates but thus far, there have been no definitive reports of lentiviral vector transgene silencing in mesenchymal stem cells. This concept of vector transgene silencing is of considerable importance where stable long-term gene expression is required. Although the present study is not a conclusive demonstration that promoter/transgene silencing or inactivation does not occur, the experimental results suggest that it is not a major factor to be considered, with respect to rat MSC transduction by the second generation VSV-G rHIV-pWPT-EF1-α-GFP-W lentiviral vector. Upon consideration of the collective data from the vector and silencing experiments, it is clear that the second generation VSV-G pseudotyped, EF1-α promoter driven construct, rHIV-pWPT-EF1-α-GFP-W, is the vector of choice for the successful and efficient *in vitro *transduction of MSCs. Modification with this particular vector facilitated sustained, persistent and high-level transgene expression in target rat MSCs, possibly due to various factors, the most likely being promoter type. Several *in vitro *studies have compared promoters in similar lentiviral vector constructs and have determined the Ef1-α promoter to be superior to other promoters tested and mediate high transgene expression levels [33]. This finding is of particular relevance to the use of MSCs for *ex vivo *transplantation in various preclinical models, where long term gene expression is required. To this end, this second generation VSV-G rHIV-pWPT-EF1-α-GFP-W lentiviral vector was chosen for all further MSC modification in this study.

In addition to assessment of transduction efficiency, we subsequently sought to examine the survival and differentiation potential of MSCs modified with this vector. As expected, a dose-response effect was achieved by transducing cells with increasing MOI, with levels approaching 100% transduction being achieved at MOI 100. In addition, we examined any toxic effect that transduction with this vector may have on cell viability and found that lentiviral vector modification of rat MSCs had no significant negative effects. Levels of cell death did not surpass ~5% of total cell population when transduced with the rHIV-pWPT-EF1-α-GFP-W vector at a high MOI of 100. This observed low level of cell death at the highest MOI measured may be in part due to a toxic effect of transgene rather than toxicity of the vector itself. GFP expression has previously been documented as being toxic in various cell types [34]. This lack of toxicity is significant and advantageous since not all vector types give the same results, for example gene transfer by adenovirus, lipid transfection and electroporation has been shown to be somewhat toxic to target cell populations [11]. It is noteworthy that, despite the integrative nature of lentivirus, modification with pWPT-EF1-α-GFP-W vector does not have any deleterious effects on fundamental MSC differentiation function. There was no significant deterioration in differentiated cell numbers between non-transduced and transduced MSC populations, clearly demonstrating that MSC uptake of the VSV-G pseudotyped lentivirus, vector DNA integration and resultant transgene expression at a high MOI does not adversely affect MSC plasticity. The same pattern has been observed with various other transgenes (data not shown). Based on these data, it is likely that other differentiation pathways would be equally unaffected by lentiviral vector modification *per se*. Indeed, several studies have similarly observed that MSC transduction with lentivirus does not negatively affect osteogenic, chondrogenic or adipogenic differentiation, and that transgene expression is retained in the differentiated cells [11,35]. This fact further supports the clinical use of these genetically modified MSCs as gene delivery vehicles or as an enhanced therapeutic treatment for diseased and damaged tissue.

This study also investigated the effect of MSC transduction at subsequent passages and demonstrated that MSC passage number is important and affects vector transduction efficiency in terms of transgene expression levels. MSC transduction was more efficient at early passage numbers and was significantly decreased at later passage numbers. This is of importance for future experiments, as transduction at earlier passage numbers ultimately results in higher levels of transgene expression. It was also determined that cryopreservation of transduced MSCs had no negative effect on transgene expression levels. This is of particular relevance to preclinical and human trials, from a logistical perspective, as it is standard practice to harvest cells, transduce, expand and freeze, ready for therapeutic use. Other gene therapy vectors are unsuitable in this respect for MSC gene delivery, as with certain vectors e.g. adenovirus, continued and sustained transgene expression is not achievable [36]. As preclinical models are designed with the eventual scale-up to human trials in mind, it is necessary to examine the transduction of animal cells compared to cells of human origin. Here, no significant difference in transgene GFP levels were observed between human and rat cells, making it possible that preclinical studies arising from rat MSCs modified with this lentiviral vector could be translated into future human studies. These findings on rHIV-pWPT-Ef1-α-GFP-W MSC transduction are important for the possible future use of modified MSC in a clinical setting.

### *In vitro* ischaemia (oxygen, serum and glucose deprivation)

In our investigation of lentivirus transduction of MSCs, we also sought to demonstrate a functional effect mediated by lentivirus vector-mediated genetic modification. For this, we primarily focused on an *in vitro *model of ischaemia, a condition of particular relevance to MSC transplantation, and several putative pro-survival genes. In our laboratory, Mylotte *et al. *demonstrated that MSCs tolerated *in vitro *conditions of hypoxia and ischaemia better than cardiomyocytes and were more vulnerable to ischaemia induced death rather than that of hypoxia [9]. This suggests that MSCs may possess the ability to survive short periods of hypoxia but not hypoxia with additional glucose and serum deprivation i.e. ischaemia. This survival capacity may however be amenable to improvement, by various gene therapy methods, thereby enhancing MSC therapeutic benefit.

In the present study, we assessed any benefit of induced therapeutic gene expression to MSCs, in an *in vitro *representation of the post-MI state, consisting of hypoxia and nutrient deprivation. Here, we found that MSCs tolerated hypoxia relatively well up to 72 h, after which survival was adversely affected in terms of cell morphology, viability, cell numbers and apoptosis. At all time points measured, exposure to ischaemia significantly reduced MSC numbers and viability and significantly increased apoptosis. Addition of the glycolytic inhibitor, 2DG, has previously been used to mimic the inhibition of glycolysis that may occur during *in vivo *episodes of ischaemia [37,38]. Thus, 2DG was incorporated into the aforementioned ischaemia conditions representing a more severe model of ischaemia. Not unexpectedly, this most rigorous of all the injury models, significantly exacerbated MSC death levels compared to hypoxia and ischaemia, suggesting that glycolysis may support MSC survival in ischaemia.

All treatments triggered cell death by apoptosis in a time dependent manner, as identified by morphology (membrane blebbing, cell and nuclear shrinkage, chromatin condensation) and caspase-3 activity. Hypoxia activated apoptotic cell death at 72 h whereas, under ischaemic conditions, apoptosis occurred earlier at 48 h as well as at 72 h. Caspase activation detected in oxygen and serum-deprived MSCs correlated with time points where the highest cell death levels were observed, suggesting that MSC death is caspase dependent. These results were consistent with findings from other researchers that have observed MSCs to be relatively resistant to hypoxia to a certain point, but sensitive to ischaemia ± 2DG [9,39]. The models of ischaemia used in this study are well established [39-42] and combine the *in vivo *ischaemia components; hypoxia, serum deprivation and glucose deprivation. As higher levels of MSC death occurred in the models that incorporated serum and glucose deprivation as well as hypoxia, it is reasonable to conclude that MSCs are more vulnerable to apoptosis as a result of nutrient and survival growth factor withdrawal rather than hypoxia alone. With this in mind, rendering the MSC more resistant to apoptosis by any means would prove beneficial for the use of transplanted MSCs in the treatment of MI.

### Induced pro-survival Gene Over-Expression

Following preliminary analysis of treatment effect, MSCs were transduced to over-express high levels of the pro-survival genes catalase, HSP27, HSP70, SOD1 and SOD3. The ability of the modified MSCs to tolerate the treatment conditions of conditions of hypoxia, ischaemia and ischaemia + 2DG, compared with wild-type MSC was subsequently examined. Transduced MSC groups were observed to be more resistant to all treatments compared to control GFP and unmodified MSC groups in terms of survival. Cell survival was assessed initially by MTT viability assay so that the most effective gene could be focused on for further study. Of the genes tested, HSP70 over-expression significantly increased MSC viability in conditions of hypoxia, ischaemia and ischaemia + 2DG. In order to ascertain the manner of cell death occurring as a result of hypoxia and ischaemia, analysis of nuclear morphology and caspase induction was performed. The occurrence of apoptosis in response to *in vitro *models of hypoxia and ischaemia has been previously reported [9,39]. We found that apoptosis was induced by hypoxia at 72 h and not unexpectedly occurred earlier in MSCs exposed to ischaemia and ischaemia + 2DG. Most importantly, we demonstrated that the induced over-expression of HSP70 prevented MSC death by apoptosis. In response to hypoxia and ischaemia, there were significantly fewer apoptotic nuclei present and significantly decreased caspase-3 activity levels in the HSP70-MSCs compared to controls. These combined data demonstrate an increased MSC survival capacity mediated by lentivirus vector-mediated HSP70 over-expression, in a clinically relevant model of ischaemia.

### MSC differentiation and oxygen deprivation

The effect of oxygen deprivation-induced stress on MSC differentiation was also assessed. Only the HSP70 gene was tested in this experiment as its over-expression had the most positive effect on MSC survival in previous experiments. Here, it was observed that none of the three oxygen deprivation treatment conditions completely diminished the ability of the MSCs to produce lipid vacuoles. This is consistent with reports that have showed MSCs retained the ability to differentiate into osteoblasts and adipocytes post *in vitro *hypoxia exposure [43-45]. A study by Lennon *et al. *demonstrated optimal MSC function at a reduced oxygen concentration of 5% [46]; this is not entirely unexpected as these oxygen levels are similar to that of *in vivo *bone marrow [47]. Although differentiation capacity was not entirely eliminated, we observed it to be notably subdued, in that numbers and incidence of differentiated MSCs were significantly reduced, most obviously with exposure to ischaemia and ischaemia + 2DG. Interestingly, HSP70-MSCs retained their functional differentiation capacity, as significantly increased numbers of differentiated cells were observed in this group. Neither did lentiviral vector-mediated HSP70 over-expression have any negative impact on MSC adipogenesis under normal conditions. The low levels of differentiation observed with ischaemic MSCs was most likely because cell numbers had decreased so considerably. Perhaps the significantly increased differentiation levels associated with the HSP70-MSC group were due to the fact that HSP70 over-expression had a beneficial effect on cell survival, thereby preserving cell number.

## Conclusions

The combination of mesenchymal stem cell therapy and gene therapy offers an integrated approach towards tissue and organ repair. The success of such a combined therapy is dependent on the optimum genetic modification of MSCs. Consequently, the present study is of particular interest and validates the choice and use of lentiviral vectors for this purpose. Here, we report that MSCs can be efficiently transduced *in vitro *to express either reporter gene or variety of therapeutic genes using a second generation lentiviral vector. This is, to our knowledge, the first study that systematically examines lentiviral vector-mediated gene modification of MSCs. Information arising from such experiments is crucial to the successful genetic modification of MSCs, which has the potential to augment their therapeutic benefit.

The integrative nature of lentiviral vectors does however raise concerns of a potential risk of insertional mutagenesis. In this regard, further investigation and clarification is required and the development of integration mapping techniques or targeted gene delivery to avoid potentially oncogenic sites has become desirable. The use of a non-integrating lentivirus vector is also an option - these vectors possess a mutated integrase gene, which results in transient gene expression. Further development in this area offers the possibility to develop a novel gene-transfer vector that does not pose the risk associated with permanent integration into host cells, yet possesses the efficiency of lentiviral vectors that is required for effective genetic modification. Despite these concerns, the value of lentivirus vectors cannot be completely discounted, as these vectors are currently unsurpassed in their efficiency of gene delivery to MSCs.

Additionally, we showed that lentivirus vector modification of MSCs can exert a functional effect through over-expression of HSP70 in an *in vitro *model of ischaemia. The use of only *in vitro *testing does however restrict what conclusions can be reached from the present study, as these findings may not translate to an *in vivo *situation. Therefore, further *in vivo *experiments based on this study are necessary for the identification of a potentially therapeutic factor for use in *ex vivo *stem cell therapy.

In conclusion, we report that lentivirus vectors are a valuable gene delivery tool and lentivirus-mediated genetic modification of MSCs has the potential to significantly augment the therapeutic benefit of MSC-based therapies.

## Abbreviations

2DG: 2-deoxyglucose; 5-aza-c: 5-azacytidine; CMV: cytomegalovirus, EF1-α: elongation factor 1 alpha; ESC: embryonic stem cell; GFP: green fluorescent protein; HDAC: histone deacetylase; HIV-1: human immunodeficiency virus-1; HSC: hematopoietic stem cell; HSP: heat shock protein; MFI: mean fluorescence intensity; MOI: multiplicity of infection; MSC: mesenchymal stem cell; PGK: phosphoglycerate kinase; SIN: self-inactivating; SOD: superoxide dismutase; TSA: trichostatin A; VSV-G: vesicular stomatitis virus glycoprotein.

## Competing interests

TOB has received education grants from Pfizer, Novartis and Merck Ltd and has received research grants from Medtronic. The other authors declare no competing interests.

## Authors' contributions

LMG produced lentiviral vectors, designed and conducted all experiments and drafted the manuscript. JMM participated in supervision and design of the study. DOT and PS participated in vector and experimental design. FB and MM participated in experimental design. TOB participated in supervision and study design and was the principal investigator. All authors carried out revisions of the manuscript and approved the final manuscript for submission.

## Supplementary Material

Additional file 1 **Transgene expression in lentivirus vector modified MSCs**. Transgene expression was demonstrated by immunostaining of (i) transduced and (ii) non-transduced samples and also by (iii) western blot for (A) catalase, (B) HSP70, (C) SOD1 and (D) SOD3. Data are representative images (scale bars 130 μm), of three independent experiments. Legend: cat = catalase: 65 kDa, H70 = HSP70: 70 kDa, HBA = Human b Actin 42 kDa, S1 = SOD1: 16 kDa, S3 = SOD3: 25.8 kDa, N/T = non-transduced rat MSCs. Click here for file
