# Supplementary figures and images for: Lentiviral vector mediated modification of mesenchymal stem cells & enhanced survival in an in vitro model of ischaemia
Source: Stem Cell Res Ther. 2011 Mar 7;2(2):12. doi: 10.1186/scrt53 (PMC3226283; doi:10.1186/scrt53)

# McGinley et al. Supplementary Data Figure 1

**A**

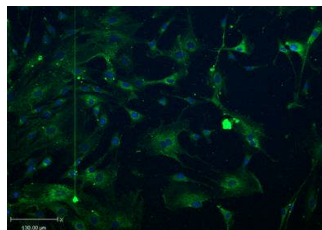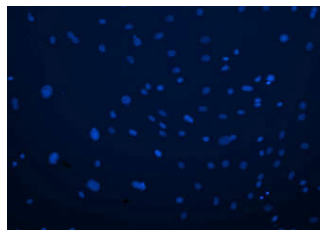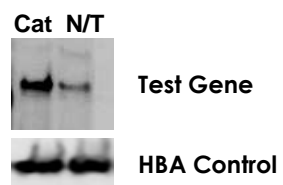

**B**

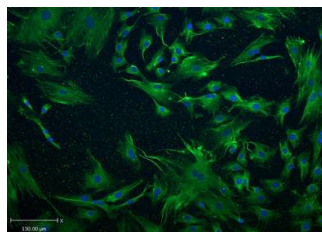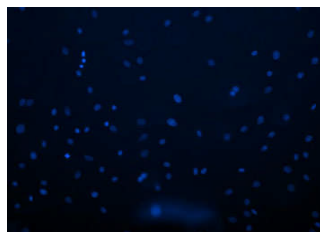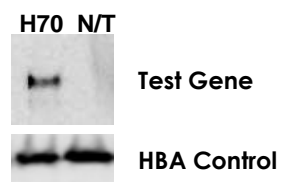

**C**

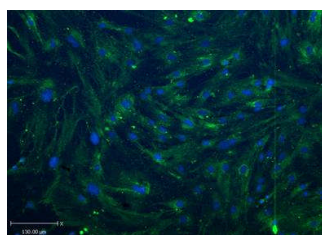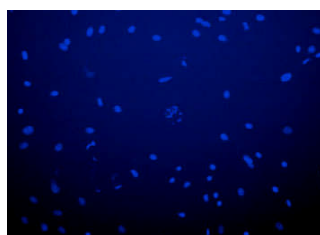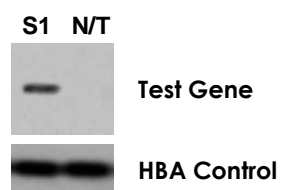

**D**

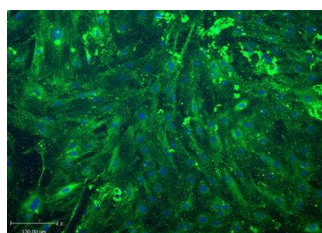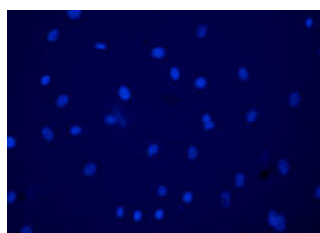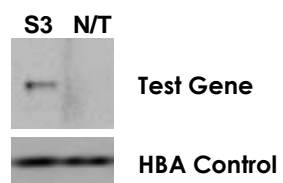

Supplement: Additional file 1 — Transgene expression in lentivirus vector modified MSCs. Transgene expression was demonstrated by immunostaining of (i) transduced and (ii) non-transduced samples and also by (iii) western blot for (A) catalase, (B) HSP70, (C) SOD1 and (D) SOD3. Data are representative images (scale bars 130 μm), of three independent experiments. Legend: cat = catalase: 65 kDa, H70 = HSP70: 70 kDa, HBA = Human b Actin 42 kDa, S1 = SOD1: 16 kDa, S3 = SOD3: 25.8 kDa, N/T = non-transduced rat MSCs. [file scrt53-S1.PDF]
